# Cellular Senescence Contributes to the Dysfunction of Tight Junctions in Submandibular Glands of Aging Mice

**DOI:** 10.1111/acel.14470

**Published:** 2025-01-09

**Authors:** Zhuo Chen, Qian‐Ying Mao, Jie‐Yuan Zhang, Yu‐Xiao Wu, Xiao‐Feng Shan, Yan Geng, Jia‐Yi Fan, Zhi‐Gang Cai, Ruo‐Lan Xiang

**Affiliations:** ^1^ Department of Physiology and Pathophysiology Peking University School of Basic Medical Sciences Beijing China; ^2^ Department of Oral and Maxillofacial Surgery Peking University School and Hospital of Stomatology & National Center for Stomatology & National Clinical Research Center for Oral Diseases & National Engineering Research Center of Oral Biomaterials and Digital Medical Devices & Beijing Key Laboratory of Digital Stomatology & NHC Key Laboratory of Digital Stomatology & NMPA Key Laboratory for Dental Materials Beijing China; ^3^ Department of Otolaryngology, Head and Neck Surgery Peking University First Hospital Beijing China; ^4^ Beijing no.161 High School Beijing China

**Keywords:** aging, cellular senescence, dental pulp stem cell‐derived exosomes, submandibular gland, tight junctions

## Abstract

The current mechanism by which aging reduces salivary secretion is unknown. This study investigates the mechanism of aging‐related submandibular (SMG) dysfunction and evaluates the therapeutic potential of dental pulp stem cell‐derived exosomes (DPSC‐exos). We found that the stimulated salivary flow rate was significantly reduced in naturally aging and D‐galactose‐induced aging mice (D‐gal mice) compared to control mice. Acinar atrophy and periductal fibrosis in SMGs and parotid glands (PGs) were observed in naturally aging and D‐gal mice, whereas sublingual glands (SLGs) had no notable alterations. We observed the accumulation of senescent cells in the SMGs, along with a decrease in claudin‐3 (Cldn‐3) expression and alterations in the distribution of Cldn1 and Cldn3. Additionally, after D‐gal‐induced senescence of SMG‐C6 cells, there was a decrease in paracellular pathway permeability, reduced expression of Cldn3 and occludin, and changes in the distribution of Cldn1, 3, 4, and 7. Furthermore, injecting DPSC‐exos into the SMGs of D‐gal mice improved stimulated salivary flow rate, reduced acinar atrophy, and decreased SA‐β‐gal activity. Our study identified that increased senescence of SMGs in aging mice can cause a decrease in salivary secretion by disrupting the expression and distribution of tight junction molecules, and injection of DPSC‐exos ameliorates SMG secretory dysfunction. These findings may provide new clues to novel therapeutic targets for aging‐related dysfunction of SMGs.

## Graphical abstract

In aging mice, the submandibular gland, among the three major salivary glands (submandibular, parotid, and sublingual glands), shows a significant increase in senescent cells. This increase disrupts tight junction structures and is closely linked to aging‐related declines in salivary secretion. Treatment with dental pulp stem cell‐derived exosomes significantly reduces senescent cell numbers in the submandibular gland and mitigates aging‐induced salivary secretion dysfunction.

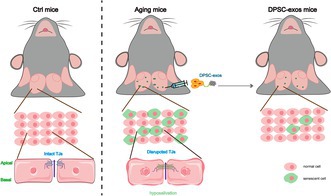

## 1 Introduction

Saliva is essential for maintaining oral health, playing a role in lubrication, taste, chewing, swallowing and initial immune defense (Roblegg, Coughran, and Sirjani 2019). Studies have shown that older people experience decreased salivary secretion, leading to symptoms such as dysphagia, increased risk of dental caries, and dysbiosis of the oral microbiota (Affoo et al. 2015; Xu, Laguna, and Sarkar 2019). Given the growing elderly population, understanding the mechanisms of salivary gland dysfunction is critical to developing treatments to improve salivary secretion and maintain oral health in older people.

Cellular senescence, characterized by irreversible cell cycle arrest, occurs due to various stimuli, including telomere dysfunction, oncogene activation, and persistent DNA damage (L. Zhang et al. 2022). Increasing research suggests a strong link between the excessive accumulation of senescent cells and age‐related diseases like Alzheimer's disease (Bussian et al. 2018), osteoarthritis (Jeon et al. 2017), atherosclerosis (Childs et al. 2016) and diabetes (Aguayo‐Mazzucato et al. 2019). The accumulation of senescent cells, particularly those positive for p16^Ink4a^, is associated with inflammatory responses and reduced lifespan. Conversely, the elimination of these cells can attenuate tissue dysfunction and improve health (Baker et al. 2016). These studies highlight the critical role of cellular senescence in aging and age‐related functional impairments. However, the specific mechanisms that cause decreased salivary secretion in older people remain unclear, and whether this decrease is related to the increase of senescent cells in the salivary glands has not been reported.

Tight junctions, cell‐to‐cell adhesion complexes located at the apical regions of adjacent epithelial/endothelial cells, dynamically regulate material transport through the paracellular pathway, playing a crucial role in saliva secretion (Baker 2016; Zhang, Wu, and Yu 2013). Recent researches have shown that dysfunction of tight junctions contributes to abnormalities in salivary secretion in diseases such as diabetes mellitus (Huang et al. 2020), Sjögren syndrome (Zhang et al. 2016) and IgG4‐related diseases (Min et al. 2020). Numerous studies have demonstrated significant alterations in tight junctions in various tissues and organs during aging, such as the skin, gastrointestinal tract, and blood–brain barrier (BBB). These findings suggest that tight junction dysfunction in older individuals compromises barrier integrity and leads to functional impairment (Andjelkovic et al. 2023; Choi 2019; Parrish 2017). In addition, in an in vitro blood–brain barrier model using senescent endothelial cells and pericytes, there was a marked diffusion of zonula occludens‐1 (ZO‐1), occludin (Ocln) and claudin‐5 (Cldn5) from the cell membrane to the cytoplasm, significantly impairing tight junctions and barrier integrity (Yamazaki et al. 2016). These studies suggest that alterations of tight junctions are associated with cellular senescence. However, the effect of aging on tight junctions in the salivary glands has not been reported. Furthermore, whether this potential impairment is associated with cellular senescence remains uncertain.

Therefore, this study aimed to investigate the functional and morphological changes in the submandibular glands (SMGs), parotid glands (PGs) and sublingual glands (SLGs) in aging mice. The study also examined the alterations in tight junctions and cellular senescence within aging SMGs and explored their relationship. Additionally, this study evaluated the potential therapeutic effects of dental pulp stem cell‐derived exosomes (DPSC‐exos) in ameliorating aging‐related SMG secretion dysfunction. These findings provide new insights into the mechanisms behind secretory dysfunction of the SMGs in older people, with the hope of offering new therapeutic strategies and a theoretical basis for the clinical treatment of reduced saliva secretion in older people.

## 2 Materials and Methods

### 2.1 Animals

The animal research was approved by the Ethics Committee of Animal Research, Peking University Health Science Center (LA2023442), and the experimental process followed the relevant animal welfare and ethics standards. The male 8‐week‐old and 18‐month‐old C57BL/6 mice were obtained from Beijing Viton Lihua Laboratory Animal Co. All animals were housed in a standard SPF ventilated lab at 18°C–22°C, under a 12‐h light–dark cycle, with access to standard mouse chow and water ad libitum. All animals were anesthetized by inhalation of isoflurane.

Establishment of D‐galactose‐induced aging mice (D‐gal mice): According to relevant literature (Azman and Zakaria 2019), the D‐galactose‐induced aging mouse model has been widely utilized in aging research. Therefore, the D‐gal mice were used in this study. Based on previous research (Liang et al. 2020; Liao et al. 2016), we selected 8‐week‐old male C57BL/6 mice and administered intraperitoneal injections of 150 mg/kg D‐gal daily for 8 weeks to effectively induce aging characteristics in the mice. Control mice were injected intraperitoneally with the same volume of normal saline every day for 8 weeks.

DPSC‐exos treatment: Mice were anesthetized using an isoflurane gas anesthesia system. Following immobilization, a chest incision was made to expose the SMGs. D‐gal mice received in situ multipoint injections of DPSC‐exos into the SMGs using a microsyringe at a dose of 100 μg in 50 μL per mouse. The control group was administered an equivalent volume of PBS following the same procedure.

### 2.2 Stimulated Saliva Flow Rate Detection

After anesthesia, mice were injected with 10 μg/g pilocarpine intraperitoneally, waited 3 min for the drug to take effect, and then collected saliva within 10 min into the centrifuge tubes. Saliva was collected using centrifuge tubes and weighed using an analytical balance. The difference in weight before and after collection was calculated to determine the relative stimulated salivary flow rate.

### 2.3 Histological, Immunohistochemical, and Immunofluorescence Staining

H&E, Alcian blue (AB), periodic acid‐Schiff (PAS) and oil red O staining were performed according to established protocols (Huang et al. 2020). Masson's trichrome staining was performed using a Masson's trichrome staining kit (Solarbio, China) according to the manufacturer's instructions.

For immunohistochemical staining, as described previously, sections were stained with primary antibodies at 4°C overnight. Thereafter, the sections were incubated with HRP‐conjugated secondary antibodies. Five randomly selected fields from each section were captured.

For immunofluorescence staining, the sections or paraformaldehyde‐fixed cells were blocked with 1% BSA, incubated with primary antibodies at 4°C overnight, and then incubated with secondary antibodies. Nuclei were stained with DAPI. Fluorescence images were captured under a confocal microscope (Leica TCS SP8, Germany). The concentration of primary antibodies was as follows: Cldn1 (1:200, BS1063, Bioworld, USA), Cldn3 (1:200, BS1067, Bioworld), Cldn4 (1:100, BS1068, Bioworld), Cldn7 (1:200, BS1070, Bioworld), Cldn10 (1:100, BS1064, Bioworld), Ocln (1:50, 71–1500, Thermo Fisher Scientific, USA) and ZO‐1 (1:100, 40–2200, Thermo Fisher Scientific).

### 2.4 Cell Culture

The rat SMG cell line SMG‐C6, generously provided by Dr. David O. Quissell, was cultured in a humidified incubator at 37°C and 5% CO2 as previously described (Mao et al. 2023).

### 2.5 CCK‐8 Assay and SA‐β‐Gal Staining

The proliferation ability of SMG‐C6 cells was performed using the CCK‐8 according to the manufacturer's instructions (DOJINDO, Japan). SMG‐C6 cells were plated in 96‐well plates and then treated with different concentrations of D‐gal for 24 h and 48 h. CCK8 solution (10% CCK8 in serum‐free DMEM medium) was added and incubated for 2 h at 37 °C. Then, the optical density values were measured at 450 nm by EnSpire Multilabel Plate Reader (PerkinElmer, USA).

SA‐β‐gal staining was performed according to the manufacturer's instructions (Cell Signaling Technology, USA). Briefly, cells or freshly frozen SMGs, PGs and SLGs sections (7 μm) were fixed with the fixative solutions and incubated overnight in the β‐Galactosidase staining solution (pH 6.0) at 37°C. The results were obtained through a light microscope (EVOS FL AUTO, USA), and SA‐β‐gal positive cells were stained blue.

### 2.6 Western Blot Analysis

Total proteins were extracted from SMG tissues and SMG‐C6 cells by RIPA lysis buffer (Thermo Fisher Scientific) with 1% cocktail (Mei5 Biotech, China), and the concentration was determined using the bicinchoninic acid assay (Solarbio, China). Equal proteins (20 μg) were separated by 10% and 12% SDS–PAGE and transferred onto polyvinylidene fluoride membranes. The membranes were blocked with 5% non‐fat milk to prevent nonspecific binding of the antibody and then incubated with primary antibodies at 4°C overnight. The HRP‐conjugated secondary antibodies were incubated for 1 h at room temperature. Target proteins were detected using an enhanced chemiluminescence reagent (Biodragon, China). Image J software was used to quantify the density of bands. Primary antibodies used are listed below: p53 (1:1000, #2527, Cell Signaling Technology), Cldn1 (1:1000, BS1063, Bioworld), Cldn3 (1:1000, BS1067, Bioworld), Cldn4 (1:1000, BS1068, Bioworld), Cldn7 (1:1000, BS1070, Bioworld), Cldn10 (1:1000, BS1064, Bioworld), Ocln (1:1000, 71–1500, Thermo Fisher Scientific) and β‐actin (1:4000, #4970, Cell Signaling Technology).

### 2.7 Reverse Transcription PCR and Real‐Time PCR

According to the manufacturer's instructions, total RNAs were extracted from SMG tissues and SMG‐C6 cells using Trizol (Invitrogen, USA). cDNA was synthesized from 2 μg RNA using 5 × All‐In‐One RT MasterMix (Vazyme, China) and amplified using 2 × SYBR Green qPCR Master Mix (Genstar, China) under a PikoReal Real‐Time PCR System (Thermo Fisher Scientific). The primers used in this study are listed in Table S1.

### 2.8 Transepithelial Electrical Resistance (TER) Measurement and Paracellular Permeability Assay

SMG‐C6 cells were cultured on Corning Transwell Filters (6.5 mm diameter, 0.4 μm pore size, USA) at a seeding density of 2.5 × 10^4^ cells/cm^2^. Within 5–7 days, the cells formed a confluent monolayer. Transepithelial electrical resistance (TER) was measured at 37°C using an epithelial volt/ohm meter EVOM2 (World Precision Instruments, USA).

After forming a confluent monolayer, 4 or 40 kDa FITC‐dextran was added to the lower chamber and incubated for 3 h. The apical solution was then collected, and the fluorescence intensity was determined using an EnSpire Multilabel Plate Reader (PerkinElmer).

### 2.9 Exosome Isolation, Identification, and Tracking

DPSCs were cultured in complete medium with 10% FBS. After 24 h, the medium was replaced with 10% exosome‐depleted FBS, and cells were cultured for a further 48 h. The supernatant was collected, centrifuged at 800 g for 5 min and 2000 g for 10 min at 4°C to remove cells and debris, and filtered through a 0.22 μm sterile filter. The filtrate was ultracentrifuged at 100,000 g for 2 h at 4°C, washed with PBS, and resuspended in 150 μL PBS (Du et al. 2023). DPSC‐exos were labeled with the membrane dye 5 μM DiR at 37°C for 30 min, followed by ultracentrifugation at 100,000 g for 2 h to remove excess dye. The pellet was resuspended in cold PBS. For identification, DPSC‐exos were examined with a transmission electron microscope (JEM1400 PLUS, Japan) and exosomal markers were identified by western blot analysis. Furthermore, the size distribution and particle concentration were determined by nanoparticle tracking analysis with a NanoSight NS300 (Malvern, UK). The distribution of DPSC‐exos in mice was examined using the IVIS SPECTRUM imaging system (PerkinElmer).

### 2.10 Statistical Analysis

Data are presented as mean ± SEM. Statistical analyses involved Student's *t*‐test for comparisons between two groups and one‐way analysis of variance (ANOVA) for multiple group comparisons, with Bonferroni's test for further analysis. *p* < 0.05 was considered statistically significant. The statistical analyses were performed using GraphPad Prism 8.0.

## 3 Results

### 3.1 Decreased Salivary Secretion in Naturally Aging and D‐Gal Mice

**FIGURE 1:**
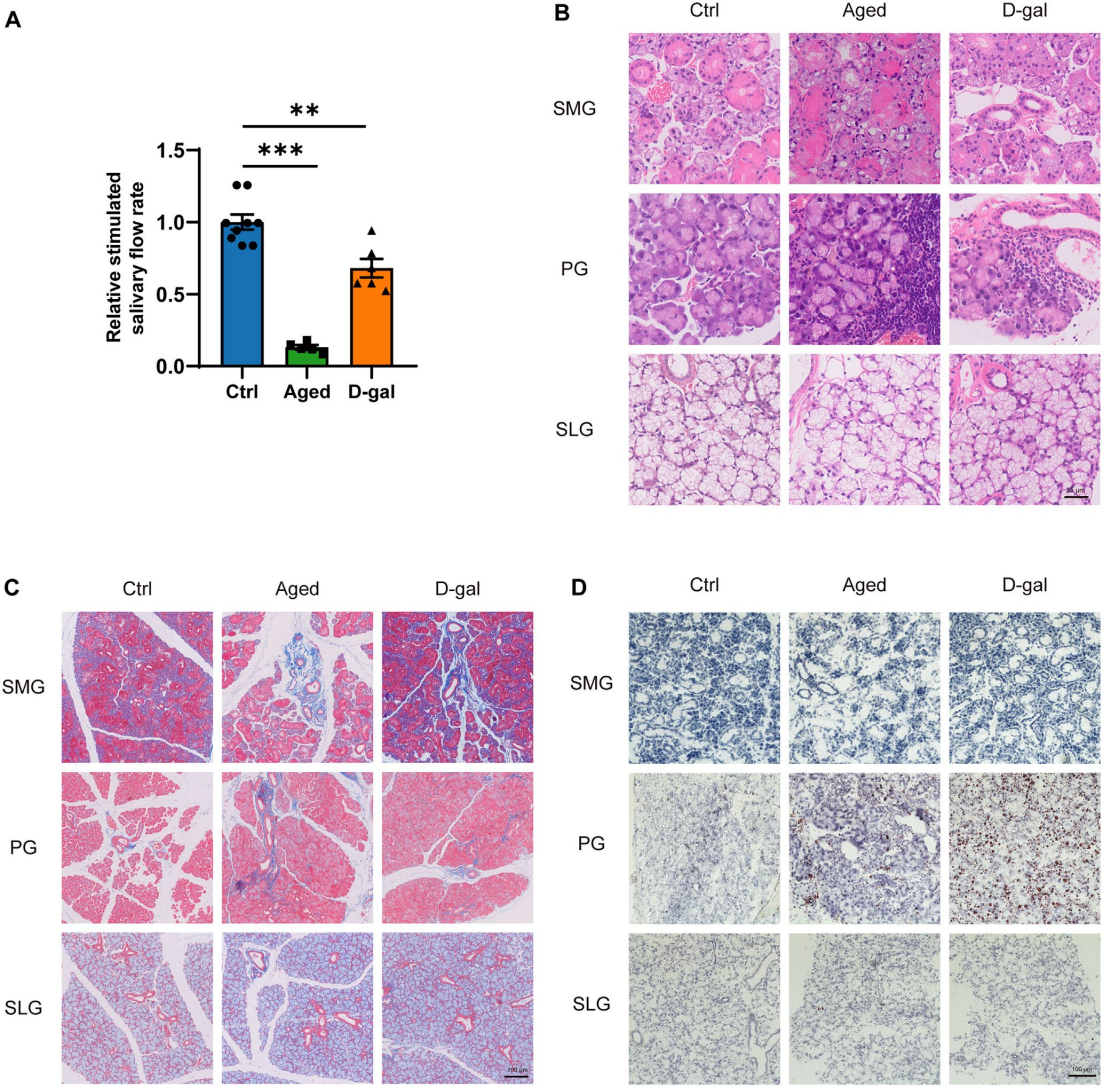
The function and structure of submandibular, parotid, and sublingual glands in control, naturally aging, and D‐galactose‐induced aging mice. (A) Relative stimulated salivary flow rate of ctrl, aged, and D‐gal group. (B) H&E staining of SMGs, PGs, and SLGs among ctrl, aged, and D‐gal group. Bar: 25 μm. (C) Masson staining of SMGs, PGs, and SLGs among ctrl, aged, and D‐gal group. Bar:100 μm. (D) Oil Red O staining of SMGs, PGs, and SLGs among ctrl, aged, and D‐gal group. Bar: 100 μm. Aged, naturally aging mice; Ctrl, control mice; D‐gal, D‐galactose‐induced aging mice; PGs, parotid glands; SLGs, sublingual glands; SMGs, submandibular glands. Data are presented as mean ± SEM; *N* = 5–9, **p < 0.01, ***p < 0.001 compared with control group.

We performed intraperitoneal injections of pilocarpine to detect relative stimulated salivary flow rate in mice and to assess changes in salivary gland secretory function in naturally aging and D‐gal mice. Our results showed a significant reduction in stimulated salivary flow rate in both naturally aging and D‐gal mice compared to control mice (Figure 1A).

### 3.2 Impaired Histological Structure of SMGs and PGs in Naturally Aging and D‐Gal Mice

Subsequently, we assessed histological changes in the SMGs, PGs, and SLGs of naturally aging and D‐gal mice. H&E staining showed that compared to the control mice, both naturally aging and D‐gal mice exhibited significant acinar atrophy and cytoplasmic vacuolization in the SMGs. In the PGs, acinar atrophy was accompanied by significant lymphocyte infiltration. In contrast, the SLGs showed no significant histological changes (Figure 1B). Masson's trichrome staining showed fibrous collagen deposition and connective tissue hyperplasia around the ducts of the SMGs and PGs in naturally aging and D‐gal mice, with no significant changes observed in the SLGs (Figure 1C). Oil Red O staining indicated that lipid deposition was present only in the PGs of naturally aging and D‐gal mice compared to control mice, with no lipid deposition observed in the SMGs and SLGs (Figure 1D). Alcian Blue and Periodic Acid‐Schiff staining showed that in both naturally aging and D‐gal mice, the expression of neutral and acidic mucins in the SMGs, PGs, and SLGs was not significantly different from that observed in the control mice (Figure S1).

### 3.3 Accumulation of Senescent Cells of SMGs in Naturally Aging and D‐Gal Mice

**FIGURE 2:**
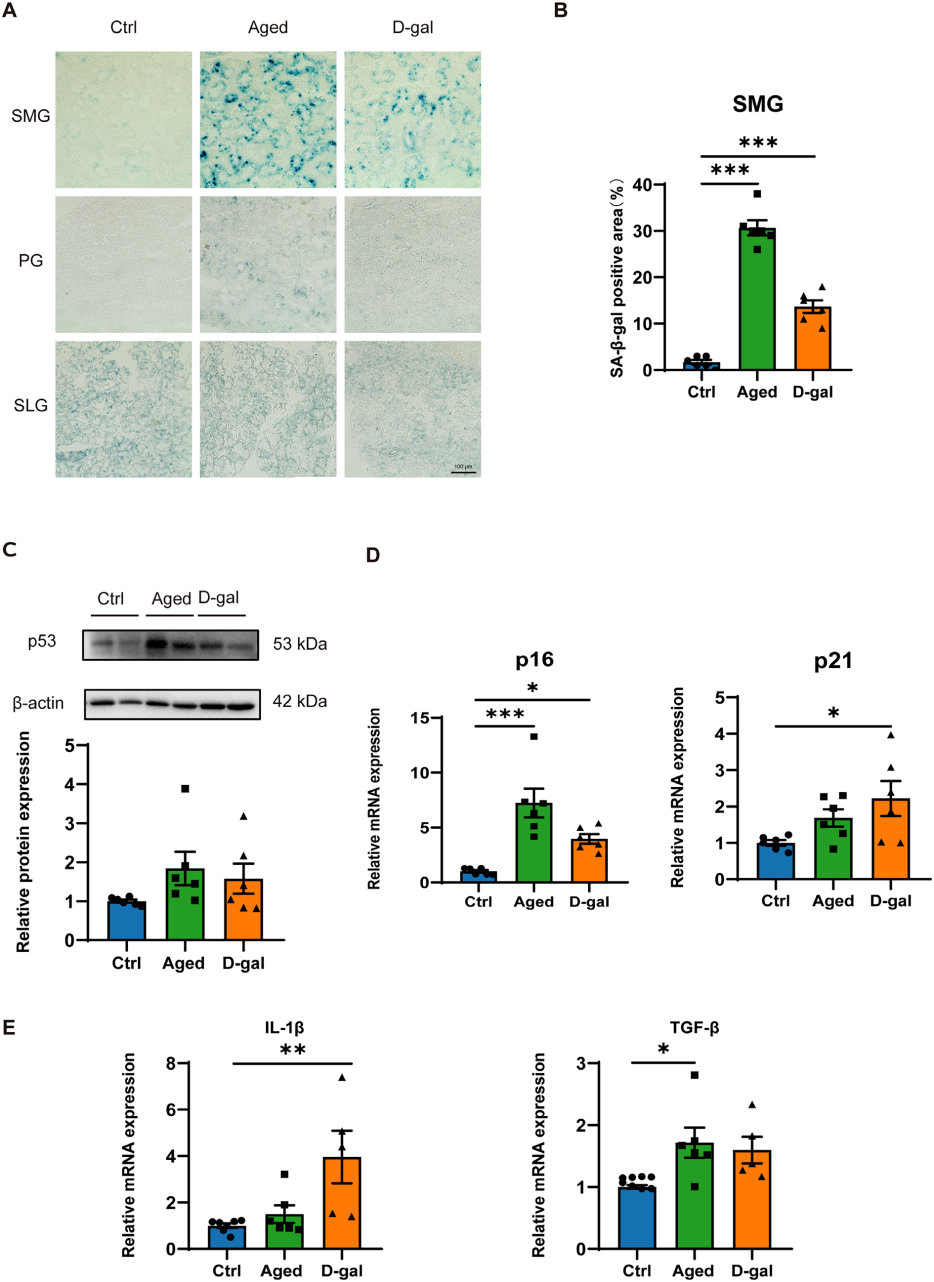
Accumulation of senescent cells of submandibular glands in naturally aging and D‐galactose‐induced aging mice. (A) SA‐β‐gal staining of the SMGs, PGs, and SLGs of ctrl, aged, and D‐gal group (B) Quantitative analysis of the SA‐β‐gal positive area in SMGs. Bar: 100 μm. (C) Western blot analysis of p53 in SMGs of ctrl, aged, and D‐gal group. (D) The mRNA expression of p16 and p21 in SMGs of ctrl, aged, and D‐gal group. (E) The mRNA expression of IL‐1β and TGF‐β in SMGs of ctrl, aged, and D‐gal group. Aged, naturally aging mice; Ctrl, control mice; D‐gal, D‐galactose‐induced aging mice; PGs, parotid glands; SLGs, sublingual glands; SMGs, submandibular glands. Data are presented as mean ± SEM; *N* = 5–8, *p < 0.05, **p < 0.01, ***p < 0.001 compared with control group.

There is increasing evidence that cellular senescence plays a pivotal role in aging. SA‐β‐gal staining is a widely used method for detecting cellular senescence. Interestingly, compared to controls, a significant increase in SA‐β‐gal activity was observed in the SMGs of both naturally aging and D‐gal mice. In contrast, no significant change in β‐gal activity was observed in the PGs and SLGs (Figure 2A,B). We also assessed multiple markers of cellular senescence, such as p53, p16, p21, and senescence‐associated secretory phenotype (SASP) factors such as IL‐1β and TGF‐β. The protein expression of p53 showed an upward trend in both naturally aging and D‐gal mice compared to controls (Figure 2C). Compared to controls, p16 mRNA expression was significantly increased in both naturally aging and D‐gal mice, whereas p21 mRNA expression was significantly increased only in D‐gal mice (Figure 2D). Analysis of SASP factors revealed a significant increase in IL‐1β mRNA in D‐gal mice and a marked increase in TGF‐β mRNA in naturally aging mice (Figure 2E). These data indicate that the SMGs are predominantly affected by aging among the three salivary glands, with a marked accumulation of senescent cells in both naturally aging and D‐gal mice. Therefore, the following research focused on the SMGs.

### 3.4 The Changes in Tight Junction Protein Expression and Distribution in SMGs of Naturally Aging and D‐Gal Mice

**FIGURE 3:**
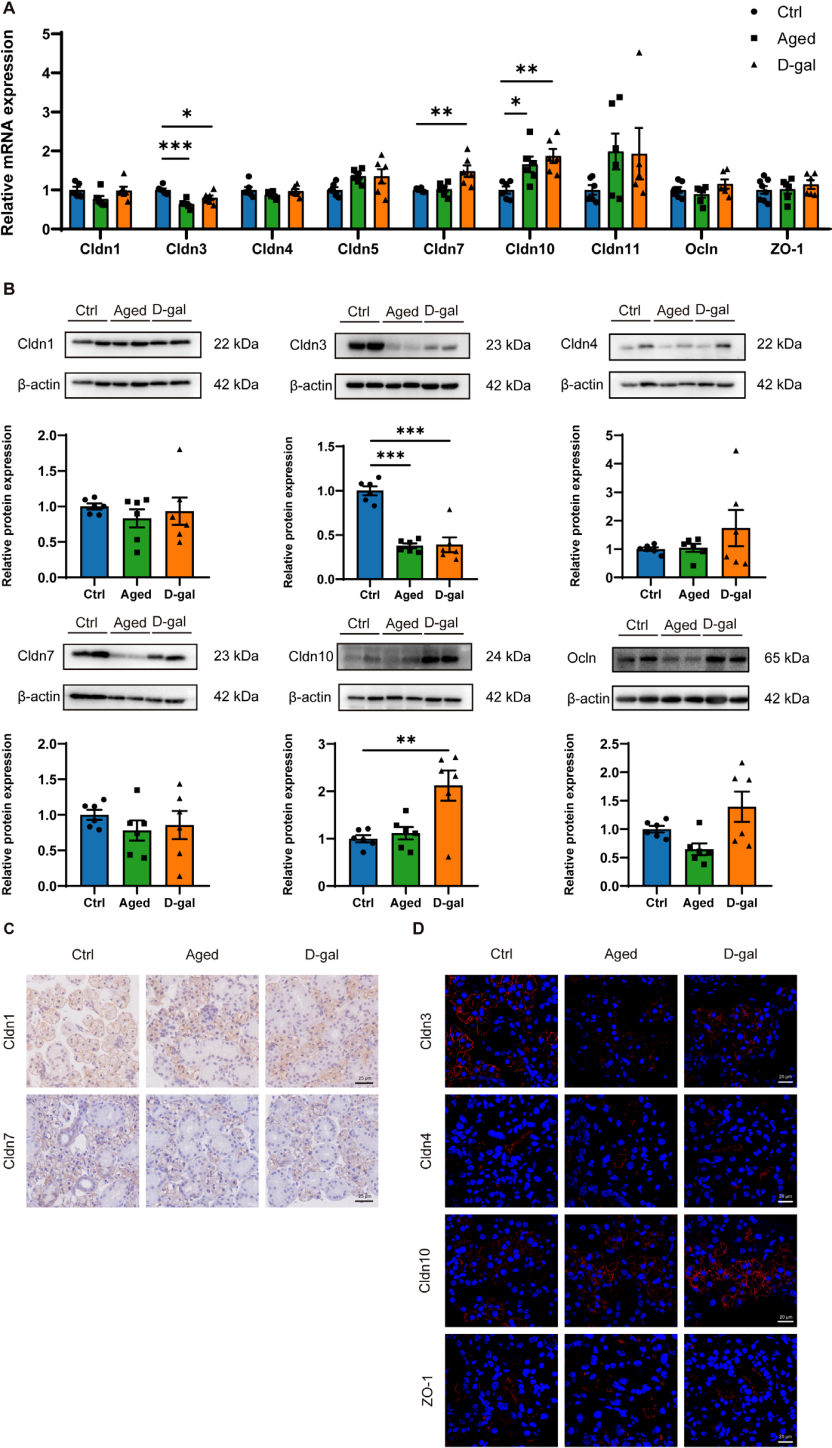
Expression and distribution of tight junction proteins in submandibular glands of control, naturally aging and D‐galactose‐induced aging mice. (A) The mRNA levels of Cldn1, Cldn3, Cldn4, Cldn5, Cldn7, Cldn10, Cldn11, Ocln, and ZO‐1 among the ctrl, old, and D‐gal group. (B) Western blot analysis of Cldn1, Cldn3, Cldn4, Cldn7, cldn10, and Ocln protein levels among the ctrl, aged, and D‐gal group. (C) Immunohistochemical staining of Cldn1 and Cldn7 in SMGs of the ctrl, aged, and D‐gal group. (D) Immunofluorescence staining of Cldn3, Cldn4, Cldn10, and ZO‐1 in SMGs of the ctrl, aged, and D‐gal group. Aged, naturally aging mice; Cldn, claudin; Ctrl, control mice; D‐gal, D‐galactose‐induced aging mice; Ocln, occludin; SMGs, submandibular glands; ZO‐1, zonula occludens‐1. Data are presented as mean ± SEM; *N* = 4–8, *p < 0.05, **p < 0.01, ***p < 0.001 compared with control group.

To elucidate the mechanisms of secretory dysfunction in the SMGs of naturally aging and D‐gal mice, we assessed the expression and distribution changes of tight junction proteins. Compared to control mice, the expression of Cldn3 mRNA was significantly decreased, while Cldn10 mRNA significantly increased in the SMGs of both naturally aging and D‐gal mice. The expression of Cldn7 mRNA was higher in naturally aging mice compared to control mice but did not change significantly in D‐gal mice. Cldn1, Cldn4, Cldn5, Cldn11, Ocln, and ZO‐1 mRNA expression showed no significant changes in the SMGs of naturally aging and D‐gal mice (Figure 3A). At the protein level, compared to the control group, Cldn3 expression was reduced in both naturally aging and D‐gal mice. Cldn10 expression showed no significant increase in naturally aging mice but was elevated in D‐gal mice. No significant changes were observed in the expression of Cldn1, Cldn4, Cldn7, or Ocln in either naturally aging or D‐gal mice (Figure 3B). Immunohistochemistry results showed that in the SMGs of naturally aging and D‐gal mice, the distribution of Cldn1 was dispersed within the cytoplasm, while the distribution of Cldn7 remained unchanged (Figure 3C). Immunofluorescence staining revealed that the distribution of Cldn3 on acinar cell membranes in naturally aging and D‐gal mice became discontinuous and scattered, significantly reducing fluorescence intensity. The fluorescence intensity of Cldn10 was increased in both naturally aged mice and D‐gal‐treated mice compared to the controls. No significant differences existed in the distribution of Cldn4 and ZO‐1 (Figure 3D). These findings indicate that during aging, alterations in the expression and distribution of tight junction proteins Cldn3, along with Cldn1 distribution, lead to impaired tight junction integrity, resulting in dysfunction of salivary secretion in the SMGs.

### 3.5 Induced Cellular Senescence of SMG‐C6 Cells After D‐Gal Treatment

**FIGURE 4:**
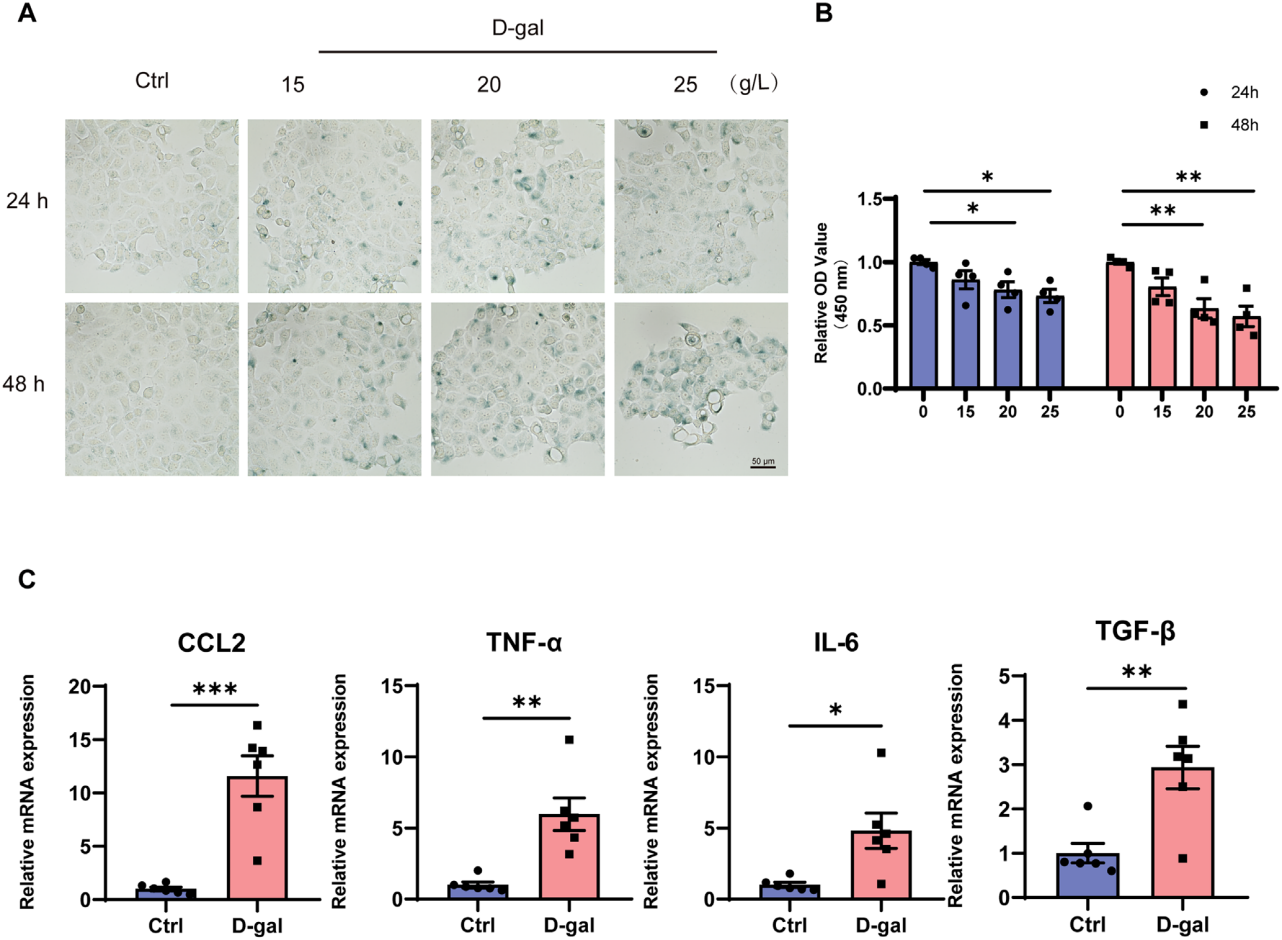
D‐galactose treatment induces cellular senescence of SMG‐C6 cells. (A) Representative images of SA‐β‐gal staining of SMG‐C6 cells after treatment with 0, 15, 20, and 25 g/L D‐gal for 24 h and 48 h. (B) The cell proliferation of SMG‐C6 cells after treatment with 0, 15, 20, and 25 g/L D‐gal for 24 h and 48 h. (C) The mRNA levels of CCL2, TNF‐α, IL‐6 and TGF‐β of SMG‐C6 cells after treatment with 20 g/L D‐gal for 48 h. D‐gal, D‐galactose. Data are presented as mean ± SEM; *N* = 4–6, *p < 0.05, **p < 0.01, ***p < 0.001 compared with control group.

To further investigate the relationship between changes in tight junction proteins and cellular senescence, we treated SMG‐C6 cells with D‐gal, a widely used agent to induce cellular senescence. SA‐β‐gal staining showed that SA‐β‐gal activity increased with higher concentrations and longer treatment times (Figure 4A). We also assessed cell proliferation using the CCK‐8 assay, which showed a significant reduction in cell proliferation ability with increasing D‐gal concentration and treatment time (Figure 4B). Accordingly, a condition of 20 g/L D‐gal for 48 h was optimal for inducing cellular senescence in SMG‐C6 cells. We further investigated SASP factors and found increased expression of CCL2, TNF‐α, IL‐6, and TGF‐β expression in SMG‐C6 cells after 48 h of treatment with 20 g/L D‐gal (Figure 4C).

### 3.6 Decreased Paracellular Pathway Permeability of SMG‐C6 Cells After D‐Gal Treatment

**FIGURE 5:**
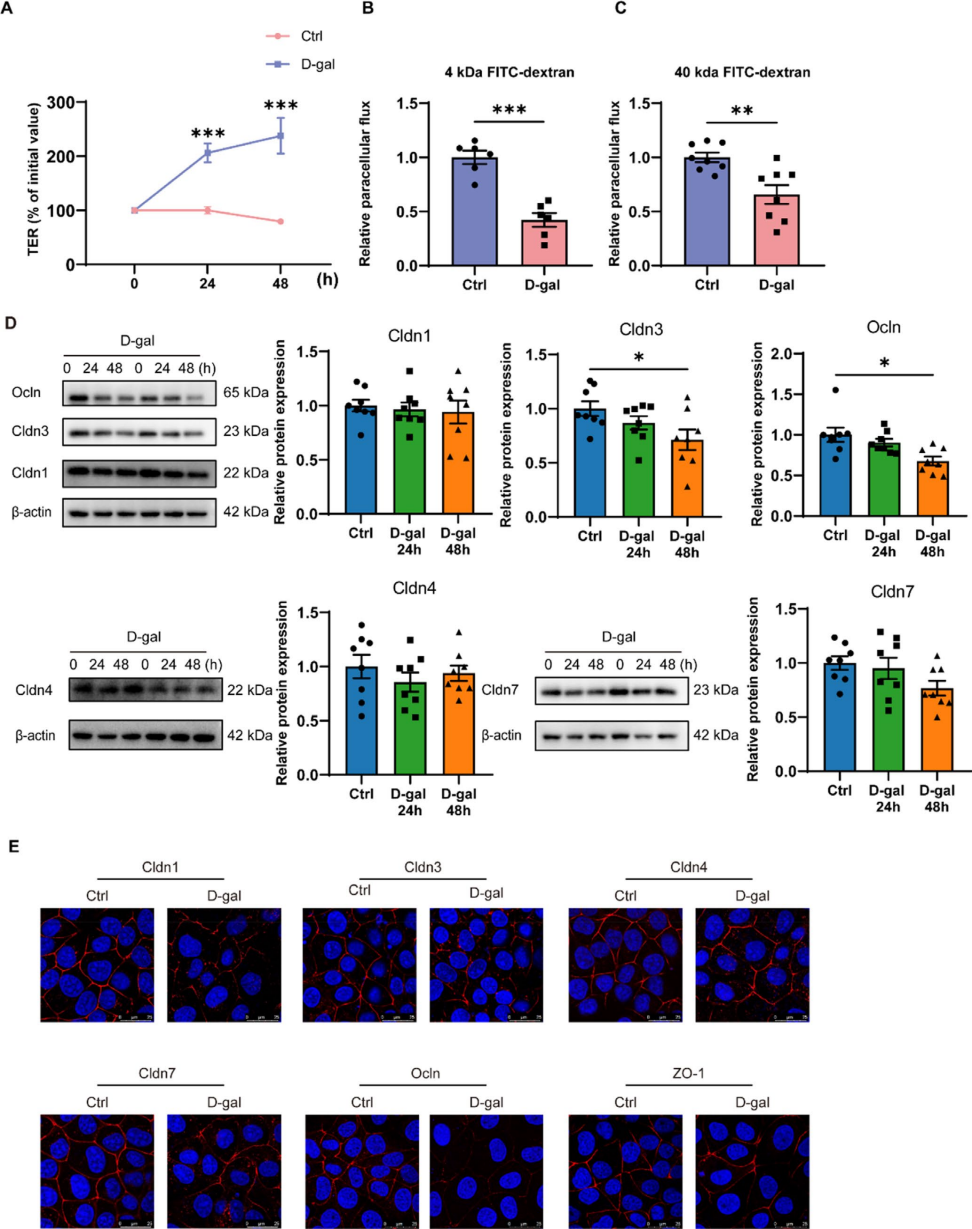
Decreased paracellular permeability and alterations in tight junctions in SMG‐C6 cells after D‐galactose treatment. (A) Transepithelial electrical resistance (TER) measurement of SMG‐C6 cells after treatment with 20 g/L D‐gal for 24 h and 48 h. (B, C) The paracellular flux assay for 4 and 40 kDa FITC‐dextran of SMG‐C6 cells after treatment with 20 g/L D‐gal for 48 h. (D) Western blot analysis of Cldn1, Cldn3, Cldn4, Cldn7, and Ocln protein levels of SMG‐C6 cells after treatment with 20 g/L D‐gal for 48 h. (E) Immunofluorescence staining of Cldn1, Cldn3, Cldn4, Cldn7, Ocln, and ZO‐1 of SMG‐C6 cells after treatment with 20 g/L D‐gal for 48 h. Cldn, claudin; Ocln, occludin; D‐gal, D‐galactose; ZO‐1, zonula occludens‐1. Data are presented as mean ± SEM; *N* = 4–6, *p < 0.05, **p < 0.01, ***p < 0.001 compared with control group.

TER provides a quantitative assessment of tight junction integrity in vitro. Due to its inability to cross the cell membrane and its availability in varying molecular weights, dextran is an ideal marker for assessing the permeability of the paracellular pathway. After inducing senescence in SMG‐C6 cells with D‐gal, we observed an increase in TER values and a decrease in permeability to 4 and 40 kDa dextran (Figure 5A–C). These results suggest that D‐gal treatment decreases the permeability of the SMG‐C6 cells' paracellular pathway.

### 3.7 The Changes in Tight Junction Proteins Expression and Distribution of SMG‐C6 Cells After D‐Gal Treatment

Next, we examined the effects of D‐gal on the expression of tight junction proteins in vitro. Western blot analysis revealed that after 48 h of 20 g/L D‐gal treatment, the expression of Cldn3 and Ocln was significantly decreased compared to the control group, whereas the expression of Cldn1, Cldn4, and Cldn7 remained unchanged (Figure 5D). Immunofluorescence staining showed a discontinuous distribution of Cldn1, Cldn3, and Cldn7 on the cell membrane and a significant reduction in Ocln fluorescence intensity. In contrast, the distribution of Cldn4 and ZO‐1 showed no significant change (Figure 5E). These results demonstrate that the expression and distribution of tight junction proteins are affected by D‐gal‐induced cellular senescence in SMG cells.

### 3.8 Improvement of SMG Secretion Dysfunction in D‐Gal Mice by DPSC‐Exos

**FIGURE 6:**
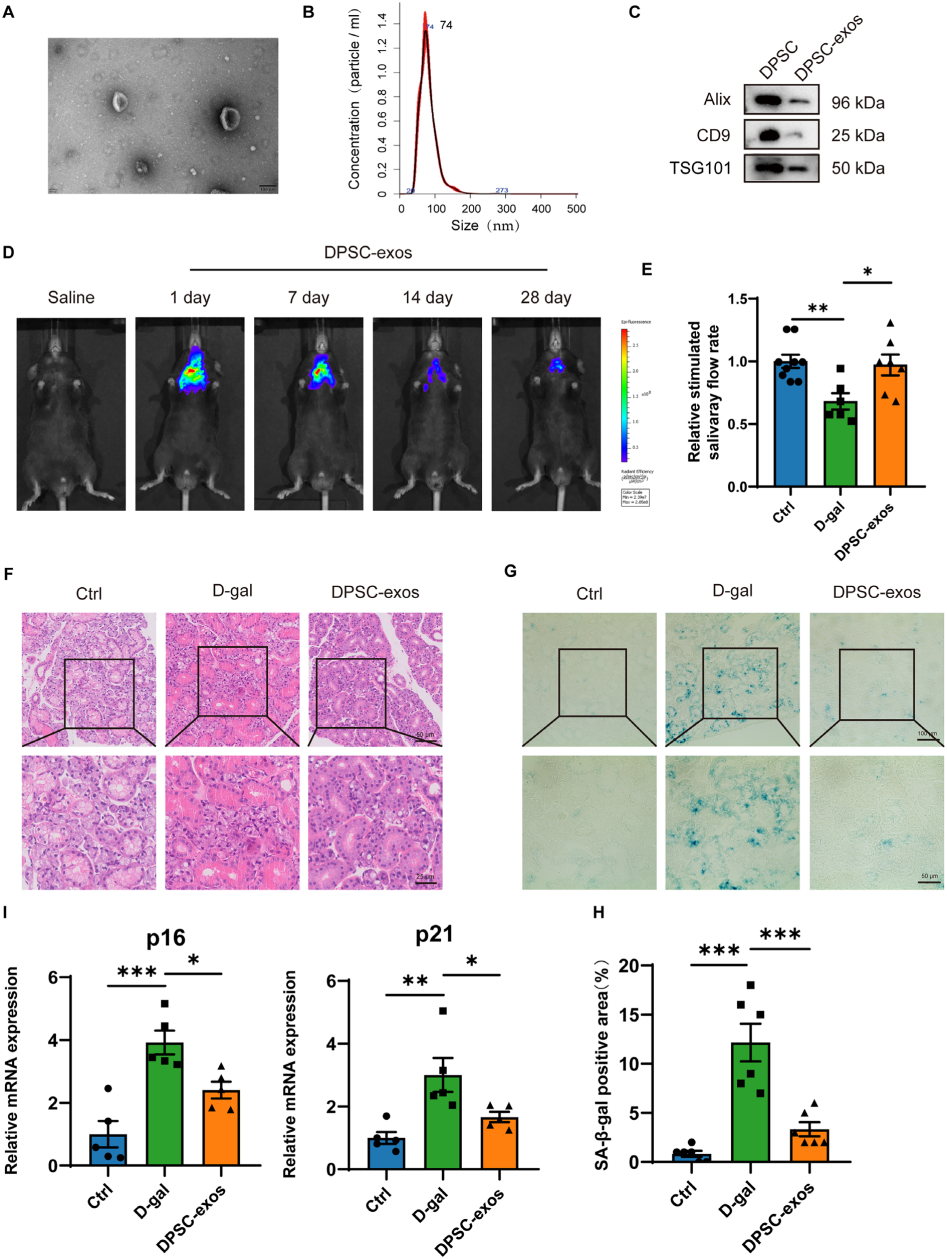
Identification and tracking of DPSC‐exosomes and improvement of dysfunction of submandibular glands by DPSC‐exos. (A) Transmission electron microscopic image of DPSC‐exos. Bar: 100 nm. (B) Nanoparticles tracking analysis of the size distribution and particle concentration of DPSC‐exos. (C) The expression of the biomarkers of DPSC‐exos, including Alix, CD9, and TSG101. (D) Bioluminescence was detected on the 1st, 7th, 14th, and 28th after the injection of Dir‐labeled DPSC‐exos. (E) Relative stimulated salivary flow rate of ctrl, D‐gal, and DPSC‐exos group. (F) H&E staining of SMGs of ctrl, D‐gal, and DPSC‐exos group. Upper bar: 50 μm, lower bar: 25 μm. (G) SA‐β‐gal staining of the SMGs of ctrl, D‐gal, and DPSC‐exos group. (H) Quantitative analysis of the SA‐β‐gal positive area in SMGs. (I) The mRNA expression of p16 and p21 in SMGs of ctrl, D‐gal and DPSC‐exos group. Upper bar: 100 μm, lower bar: 50 μm. Ctrl, control mice; D‐galactose‐induced aging mice; DPSC‐exos, dental pulp stem cell‐derived exosomes; SMGs, submandibular glands. Data are presented as mean ± SEM; *N* = 5–9, *p < 0.05, **p < 0.01, ***p < 0.001 compared with control group.

Exosomes have demonstrated therapeutic potential across various fields; we investigated whether DPSC‐exos could ameliorate aging‐related dysfunction of SMGs. DPSC‐exos were isolated and characterized, exhibiting classic cup‐shaped or sphere‐shaped microstructure with a mean diameter of 74.2 ± 2.6 nm (Figure 6A,B), and they expressed the exosome‐associated proteins Alix, CD9, and TSG101 (Figure 6C). We subsequently injected 100 μg of DiR‐labeled DPSC‐exos into the SMGs of D‐gal mice. Bioimaging techniques demonstrated that DiR‐labeled DPSC‐exos were distributed around the neck and precisely in the SMGs. The positive signal was strong on the 1st and 7th day of injection and gradually decreased on the 14th day (Figure 6D). After 8 weeks of DPSC‐exos treatment of D‐gal mice, evaluation of the stimulated salivary flow rate showed an increased secretion capacity in D‐gal mice (Figure 6E). H&E staining demonstrated morphological improvements in the SMGs, including alleviated acinar atrophy and a more organized acinar cell arrangement (Figure 6F). Additionally, SA‐β‐gal staining showed a marked decrease in positive staining within the SMGs of D‐gal mice (Figure 6G,H) and a significant decrease in the expression of both p16 and p21 mRNA (Figure 6I). These results suggest that DPSC‐exos can alleviate the dysfunction of aging‐related SMGs.

## 4 Discussion

In the present study, we found a reduction in salivary secretion in both naturally aging and D‐gal mice, with significant histological alterations in SMGs and PGs but not SLGs. Notably, our results demonstrated a significant accumulation of senescent cells in the SMGs, while no such accumulation was detected in the PGs or SLGs. We further found that the expression and distribution of tight junctions are changed in aging SMGs. Moreover, D‐gal‐induced senescence in SMG‐C6 cells led to impaired tight junction function. These findings suggest that accumulation of senescent cells in SMGs may affect impaired tight junctions, particularly abnormal expression and distribution of Cldn3 and abnormal distribution of Cldn1, thereby contributing to secretory dysfunction. Additionally, we found that DPSC‐exos can alleviate the dysfunction of aging‐related SMGs.

Mouse models of aging are divided into natural aging and accelerated aging. The latter is induced by methods such as hydroxyurea, radiation, and D‐gal. The D‐gal‐induced aging model is preferred for aging studies because of its practicality and similarity to natural aging (Cai, Wu, and Huang 2022). Therefore, we selected naturally aging and D‐gal mice to study the effects of aging on the salivary glands and the mechanisms involved. Several studies have reported degenerative changes in the salivary glands with age, including decreased epithelial volume and increased fat and connective tissue (Miyagi et al. 2019; Sørensen et al. 2014; Toan and Ahn 2021). However, the effects of aging on the three major salivary glands‐SMGs, PGs, and SLGs‐have not been extensively studied. Our research studied the functional and histological changes in these glands of aging and D‐gal mice. We observed significant atrophy of the SMGs and PGs acinar cells in naturally aging and D‐gal mice, with a marked increase in periductal connective tissue compared to controls. In contrast, the SLGs showed no significant change. Lipids deposit only in the PGs but not in the SMGs and SLGs. Our study revealed histological disruption of SMGs and PGs in naturally aging and D‐gal mice and differences between glands.

Increasing evidence suggests cellular senescence plays a critical role in age‐related structural and functional impairments (Calcinotto et al. 2019; Mohamad Kamal et al. 2020). Notably, transplanting a small number of senescent cells into young, healthy animals can alleviate age‐related dysfunction (Xu et al. 2017), while selectively removing these senescent cells can slow the progression of age‐related diseases (Xu et al. 2018). For example, targeting and eliminating senescent cells can alleviate the symptoms of post‐traumatic osteoarthritis, reduce pain, and promote cartilage development (Jeon et al. 2017). We found in naturally aging and D‐gal mice senescent cells accumulate in the SMGs, while there is no noticeable accumulation in the PGs and SLGs. In addition, we examined SASP factor IL‐1β and TGF‐β expression and observed inconsistent expression in naturally aging and D‐gal mice. Naturally aging mice probably experience a broader range of changes beyond cellular senescence, including genomic instability, epigenetic modifications, and stem cell exhaustion (López‐Otín et al. 2023). These complex and multifaceted differences may contribute to the observed differences in IL‐1β and TGF‐β mRNA expression between the two models. These results suggest cellular senescence may contribute to secretory dysfunction. Similarly, the SAM‐P1 mouse model also showed increased p16Ink4a expression in the SMGs but not in the PGs (Miyagi et al. 2019). Furthermore, among the three major salivary glands in diabetic mice, the SMGs and PGs were more susceptible to changes, while the SLGs remained largely unaffected (Huang et al. 2020). This differential susceptibility may be due to the inherent glandular structures: the SMGs are mixed seromucous, the PGs are purely serous, and the SLGs are predominantly mucous. Previous studies have shown that acetylcholine and norepinephrine concentrations are higher in SMGs than in the other major salivary glands in mice (Murai et al. 1998), suggesting enhanced functional activity in SMGs. Additionally, compared to the PGs and SLGs, the SMGs in mice contain a higher proportion of ducts (Kondo et al. 2015) and feature unique granular convoluted tubules (GCTs). GCTs exhibit high secretory activity, producing bioactive factors such as epidermal growth factor and nerve growth factor. These cells are rich in mitochondria, providing the energy required for their secretion. Mitochondria, sensitive to aging, are particularly vulnerable to damage, with both function and structure altered during aging. Given the high mitochondrial content in GCTs and their sensitivity to aging, we hypothesize that SMG mitochondria are more prone to aging‐related damage, leading to more pronounced functional decline and structural changes compared to other salivary glands.

Tight junctions are essential for maintaining barrier integrity. With aging, damage to these junctions can impair endothelial and epithelial barriers, contributing to the development and progression of various age‐related diseases. For example, the colon of aging rats showed significantly reduced Cldn1 and Ocln expression, with elevated levels of IL‐1β and TNF‐α, contributing to an age‐related decline in intestinal epithelial barrier function (Dun et al. 2018). Amyloid β deposition in the retinal pigment epithelium of older people activates the NF‐κB pathway, reducing ZO‐1 and Ocln expression (Jo et al. 2020). This disruption increases retinal endothelial permeability, leading to exudative age‐related macular degeneration. The alteration of tight junctions in SMGs during aging has not been reported. Our findings reveal that in the SMGs of naturally aging and D‐gal mice, the expression of Cldn3 protein and mRNA decreased, as well as translocation of Cldn1 from the cell membrane to the cytoplasm, with Cldn3 distribution discontinuously along the cell membrane. However, the underlying mechanisms by which aging induces changes in tight junctions remain elusive. In an in vitro BBB model, senescent vascular cells showed impaired barrier integrity and structural alterations of tight junctions, as evidenced by discontinuous staining of Cldn5, Ocln, and ZO‐1 as well as translocation of Ocln from the cell membrane to the cytoplasm (Yamazaki et al. 2016). In the hydrogen peroxide‐induced human retinal pigment epithelium cell line ARPE‐19 senescent cell model, the collapse of the tight junction and filamentous actin networks was observed, as evidenced by an abnormal distribution of ZO‐1 showing fragmentation staining and filamentous actin showing diffuse staining (Chen et al. 2021). To further investigate whether altered tight junction structure and function are related to cellular senescence, we examined whether the structure and function of tight junctions change during cellular senescence. After D‐gal‐induced senescence in SMG‐C6 cells, there was a decrease in the permeability of the paracellular pathway, decreased expression of tight junction molecules Cldn3 and Ocln, and discontinuous distribution of Cldn1, 3, and 7 along the cell membrane. Our study demonstrated that after D‐gal‐induced senescence in SMG‐C6 cells, there was a decrease in the permeability of the paracellular pathway, decreased expression of tight junction molecules Cldn3 and Ocln, and discontinuous distribution of Cldn1, 3, and 7 along the cell membrane. Study has shown that overexpression of Cldn3 in rat type II alveolar epithelial cells reduces transcellular resistance, thereby impairing alveolar epithelial barrier function (Mitchell et al. 2011). Another study found that overexpression of Cldn3 in Madin‐Darby canine kidney epithelial cells enhanced barrier function by promoting interactions between Cldn3 and Cldn2 (Milatz et al. 2010). Study has shown that in IgG4‐related sialadenitis (IgG4‐RS), characterized by decreased levels of Cldn3, Cldn4, and ZO‐1, alongside increased levels of Cldn1 and Ocln. The disruption of the TJ complex, particularly the alterations in Cldn3 and ZO‐1, might contribute to the hyposalivation observed in IgG4‐RS patients (Min et al. 2020). These findings suggest that barrier integrity is not solely determined by the expression level of a single tight junction protein but is subject to multifaceted regulation, including its spatial distribution, interactions among Claudin family members, and associations with ZO family proteins or F‐actin. The functional diversity of tight junctions arises from these varied regulatory mechanisms, and the effects of changes in tight junction protein expression on barrier function largely depend on their interactions with other tight junction components (Van Itallie, Fanning, and Anderson 2003). Our findings revealed changes not only in the expression of Cldn3 and Ocln but also in the distribution of Cldn3, Cldn1, and Cldn7. These alterations in the expression, distribution, and interactions of tight junction molecules collectively contribute to the disruption of the tight junction complex, thereby altering paracellular permeability and regulating barrier function. The above results suggest that cellular senescence may contribute to impairment of tight junction structure and function, which may lead to aging‐related SMG secretory dysfunction, suggesting that targeting cellular senescence may offer a potential therapeutic approach to ameliorate aging‐related dysfunction of SMGs.

Mesenchymal stem cell exosomes, as a novel form of biological therapy, offer benefits such as anti‐apoptotic, anti‐inflammatory and immunoregulatory properties and have shown superior therapeutic potential in several areas (Adelipour, Lubman, and Kim 2023; Kalluri and LeBleu 2020). Studies indicate that exosomes derived from labial mesenchymal stem cells can treat Sjögren's syndrome by mediating miRNA‐125b targeting PRDM1 and inhibiting plasma cells (Xing et al. 2022); exosomes from hypoxia‐preconditioned urinary stem cells can activate the Wnt3a/GSK3β pathway, thereby inhibiting the expression of α‐SMA and c‐Kit, and effectively repair radiation‐induced salivary gland damage (Xiao et al. 2022). In addition, DPSC‐exos restore the function of salivary gland epithelial cells in NOD mice through the GPER‐mediated cAMP/PKA/CREB signaling pathway (Hu et al. 2023). To date, no studies have reported whether DPSC‐exos can alleviate aging‐related SMG dysfunction. Our research has shown that DPSC‐exos can increase the stimulated salivary flow rate in D‐gal mice, improve the histological structure and reduce the number of β‐gal positive cells of aging SMGs, suggesting that DPSC‐exos may be a promising therapeutic strategy for alleviating dysfunction in aging SMGs.

Our study has several limitations. First, our experiments were conducted on mouse salivary gland tissue instead of human counterparts. It is well known that the human and mouse salivary glands are structurally different. Human SMGs are primarily composed of serous acini, whereas mouse SMGs are composed of seromucous acini. Furthermore, male mice salivary glands exhibit a unique granular convoluted structure, so our study may not entirely apply to humans. In‐depth work on this issue will be conducted in future studies.

In summary, our study reveals a decrease in salivary secretion in both aging and D‐gal mice and histological changes in the SMGs and PGs. We observed the accumulation of senescent cells in the SMGs. Furthermore, cellular senescence may contribute to changes in tight junctions, thereby leading to aging‐related hyposalivation. This research identifies potential therapeutic targets and strategies to mitigate decreased salivary secretion in older people, thereby enhancing oral health and presenting substantial theoretical and clinical implications.

## Author Contributions

R.‐L.X., Z.‐G.C., Q.‐Y.M., and Z.C. designed the study. Z.C. and Q.‐Y.M. performed the experiments and analyzed the data. J.‐Y.Z., Y.‐X.W., X.‐F.S., Y.G., and J.‐Y.F. supported the experiments. R.‐L.X. and Z.‐G.C. secured funding and provided supervision. Z.C. drafted the manuscript, and all authors edited and finalized the manuscript.

## Conflicts of Interest

The authors declare no conflicts of interest.

## Supporting information


Data S1.

## Data Availability

The data that support the findings of this study are available from the corresponding authors upon reasonable request.
