# Supplementary material for: Cellular Senescence Contributes to the Dysfunction of Tight Junctions in Submandibular Glands of Aging Mice
Source: Aging Cell. 2025 Jan 9;24(5):e14470. doi: 10.1111/acel.14470 (PMC12074015; doi:10.1111/acel.14470)
Supplement: Supplementary file 1 — Data S1. [file ACEL-24-e14470-s001.docx]

**Article title: Cellular senescence contributes to the dysfunction of tight junctions in submandibular glands of aging mice**

Zhuo Chen^1^*, Qian-Ying Mao^2^*, Jie-Yuan Zhang^2^, Yu-Xiao Wu^2^, Xiao-Feng Shan^2^, Yan Geng^3^, Jia-Yi Fan^4^, Zhi-Gang Cai^2#^, Ruo-Lan Xiang^1#^

^1^Department of Physiology and Pathophysiology, Peking University School of Basic Medical Sciences, Beijing, China

^2^Department of Oral and Maxillofacial Surgery, Peking University School and Hospital of Stomatology & National Center for Stomatology & National Clinical Research Center for Oral Diseases & National Engineering Research Center of Oral Biomaterials and Digital Medical Devices & Beijing Key Laboratory of Digital Stomatology & NHC Key Laboratory of Digital Stomatology & NMPA Key Laboratory for Dental Materials, Beijing, China

^3^Department of Otolaryngology, Head and Neck Surgery, Peking University First Hospital, Beijing, China

^4^Beijing No.161 High School, Beijing, China

*Authors contributing equally to this article.

**Correspondence**

Ruo-Lan Xiang, Department of Physiology and Pathophysiology, Peking University School of Basic Medical Sciences, No. 38 Xueyuan Road, Haidian District, Beijing, 100191, P.R. China. Email: [xiangrl@bjmu.edu.cn](mailto:xiangrl@bjmu.edu.cn)

Zhi-gang Cai, Peking University School and Hospital of Stomatology, No. 22 Zhongguancun South Avenue, Haidian District, Beijing, 100081, P.R. China. Tel: 0086-10-82195956. Email: c2013xs@163.com


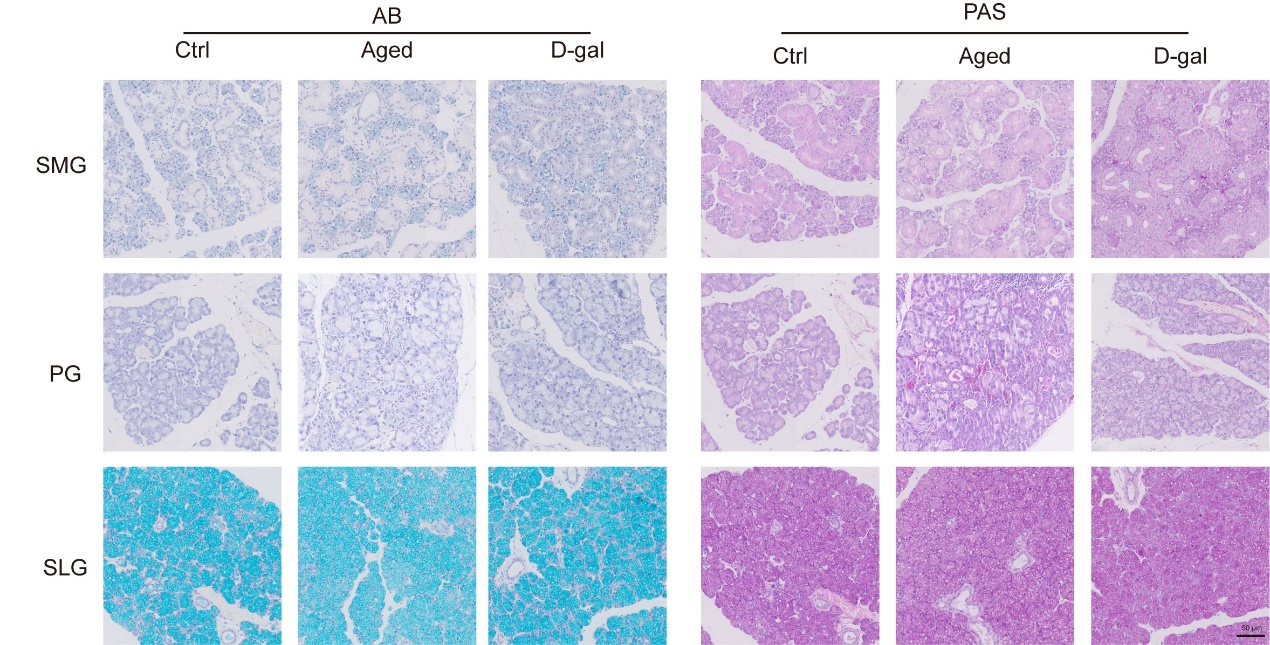


Supplementary Fig.1 Alcian blue and Periodic acid-Schiff staining of submandibular glands, parotid glands and sublingual glands among ctrl, aged and D-gal group. AB, Alcian blue; PAS, Periodic acid-Schiff; Ctrl, control mice; Aged, naturally aging mice; D-gal: D-gal-induced aging mice; SMGs, submandibular glands; PGs, parotid glands; SLGs, sublingual glands. Bar:50 μm.

Supplementary table 1. Primer sequences for RT-qPCR

| Gene | Species | Forward (5’-3’) | Reverse (5’-3’) |
| --- | --- | --- | --- |
| CCL2 | Rat | CAGGTGTCCCAAAGAAGCTGTA | CTGAAGTCCTTAGGGTTGATGC |
| TNF-α | Rat | CCTCACCCACACCGTCAG | GCAGGTCCCCCTTCTCCA |
| IL-6 | Rat | TCTGGTCTTCTGGAGTTCCG | AGCATTGGAAGTTGGGGTAGG |
| TGF-β | Rat | AGAAGTCACCCGCGTGCTAA | TCCCGAATGTCTGACGTATTGA |
| β-actin | Rat | TCGTGCGTGACATTAAAGAG | ATTGCCGATAGTGATGACCT |
| IL-1β | Mouse | GCCACCTTTTGACAGTGATG | GAAGGTCCACGGGAAAGACA |
| TGF-β | Mouse | ACTGGAGTTGTACGGCAGTG | GGGGCTGATCCCGTTGATTT |
| Cldn1 | Mouse | TATGACCCCTTGACCCCCAT | AGAGGTTGTTTTCCGGGGAC |
| Cldn3 | Mouse | TACAAGACGAGACGGCCAAG | TGGTGGGTGCATACTTGTCG |
| Cldn4 | Mouse | GATCTTGGCCTTGACGGTCTC | CTCTGGATGAACTGCGTGGTG |
| Cldn5 | Mouse | GTTAAGGCACGGGTAGCACT | GTACTTCTGTGACACCGGCA |
| Cldn7 | Mouse | TGTGGGGGAGATGACAAAGC | CATGGGCGTCAAGGGGTTAT |
| Cldn10 | Mouse | CCATGGGTAGCACGGCCTTGG | TTAGACATAGGCATTTTTATC |
| Cldn11 | Mouse | TTCCCTGCCAGGCACTAAAG | ATGCCCGAGGAAAGAGCAAA |
| Occludin | Mouse | CCATCTTTCTTCGGGTTTTCA | CTTCTGGATCTATGTACGGCTCA |
| ZO-1 | Mouse | GTAAAGCCTGGTGGTGGAACT | TCGAACCTCTACTCTACGACATG |
| p16 | Mouse | GAACTCTTTCGGTCGTACCC | CGAATCTGCACCGTAGTTGA |
| p21 | Mouse | GCAAAGTGTGCCGTTGTCTC | CGTCTCCGTGACGAAGTCAA |
| β-actin | Mouse | CCAACCGTGAAAAGATGACC | CCAGAGGCATACAGGGACAG |
